# Active Learning Strategies for Improving Dental Students' Skills in Preclinical Restorative Dentistry

**DOI:** 10.1055/s-0045-1808256

**Published:** 2025-05-07

**Authors:** Syed Zubairuddin Ahmed, Philline M. Deraney, Shazia Sadaf, Intisar Ahmad Siddiqui, Marwa Madi, Jehan AlHumaid

**Affiliations:** 1 Department of Restorative Dental Sciences, College of Dentistry, Imam Abdulrahman Bin Faisal University, Ar Rākah, Saudi Arabia; 2 Department of Curriculum and Instruction, College of Education, Imam Abdulrahman Bin Faisal University, Dammam, Saudi Arabia; 3 Department of Dental Education, College of Dentistry, Imam Abdulrahman Bin Faisal University, Dammam, Saudi Arabia; 4 Department of Preventive Dental Sciences, College of Dentistry, Imam Abdulrahman Bin Faisal University, Dammam, Saudi Arabia

**Keywords:** action research, active learning, assessment methods, dental education, flipped classroom, mixed methods, peer assessment, poster presentation, surveys

## Abstract

**Objective:**

Practical assessment in dental education is crucial for developing clinical competencies. This study investigated the effectiveness of action research (AR) and active learning strategies in improving student achievement in a preclinical restorative dentistry course among 58 third-year dental students.

**Materials and Methods:**

The intervention focused on teaching composite cavity preparation techniques through student engagement and participation. Using a mixed-methods approach, data were collected over 10 weeks through observations, surveys, poster presentations, performance evaluations, and peer assessments. Data analysis employed SPSS-20.0 for quantitative measures, including one-sample *t* -tests with a mean value of 3.6 as the cutoff point on the Likert scale for high-quality ratings (Ho: μ = 3.6, *p* ≤ 0.05).

**Results:**

The surveys demonstrated high internal reliability (Cronbach's alpha = 0.91). Students reported the highest satisfaction with topic relevance (90%) and showed significant improvement in learning outcomes through poster presentations and peer assessments ( *p* < 0.05). However, activity organization ( *p* = 0.526), clinical practice impact ( *p* = 0.072), and cross-course applicability ( *p* = 0.671) were nonsignificant. Pre- and post-test data were analyzed both statistically and descriptively to evaluate learning outcomes.

**Conclusion:**

These findings suggest that implementing AR and active learning strategies can enhance student learning and achievement in preclinical dental education, though further research is needed to optimize organizational aspects and clinical practice.

## Introduction

### Traditional Learning in Dental Education

Traditional teaching approaches were more prevalent in the past because educators wanted their students to master set material and perform well on tests. Teachers are in charge of the class in a typical classroom, where students must follow rules to maintain a positive learning environment. As the new era progresses, people's thinking becomes more active, and science and technology advance. In addition to wanting to study from textbooks, students are drawn to cutting-edge information. Modern teaching techniques are also frequently employed to raise teaching standards and give students a better education. 1

Dental education is a complex and demanding pedagogical process requiring students to attain diverse competencies over 4 to 6 years of study. 2 Conventional learning in dental education often results in unidirectional knowledge transfer and fails to identify gaps in students' understanding. 3 While traditional face-to-face lectures remain valuable in 21st-century education, 4 universities face increasing pressure to transform classroom environments into more interactive spaces. 5 6

### Benefits of Active Learning

Active learning, unlike traditional approaches, engages students in higher order thinking activities and shifts control from instructor to student, encouraging greater responsibility for learning outcomes. 7 Educational content should be made available through various methods to accommodate different learning styles and preferences, with students placed at the center of the learning process. 8

The flipped classroom approach has emerged as a particularly effective active learning method, redefining traditional teaching by moving lecture content outside the classroom and focusing on concept application during class time. 9 10 11

### Action Research in Dental Education

Action Research (AR), first conceptualized by Kurt Lewin in 1946, provides a framework for implementing educational innovations through participatory, action-based research. 12 Since its introduction to education through the Nuffield Foundation's Humanities Curriculum Project (1967–1972), AR has evolved to incorporate various methodological approaches. 13 14 The integration of mixed methods within AR frameworks enables educators to enhance student learning outcomes through systematic inquiry and data-driven modifications. 15 16

AR has proven valuable in dental education by enhancing both teaching methodologies and student outcomes. Studies have shown its effectiveness in improving clinical skills acquisition, 17 developing reflective practice among dental students, 18 and optimizing preclinical laboratory teaching. 19 AR's iterative nature aligns well with dental education's need for continuous improvement in practical skills development and theoretical knowledge integration. 20 Other implementations have demonstrated success in areas such as assessment methods, 21 problem-based learning in dentistry, 22 and clinical decision-making skills. 23 The collaborative aspect of AR has particularly benefited dental faculty development and curriculum enhancement. 24

This study assessed the impact of integrating flipped classrooms, Kahoot, and poster presentations in the clinical restorative dentistry course for third-year dental students. By combining these active learning strategies, which have not been studied before in clinical dentistry course, we aim to improve the traditional teaching environment with increased student-centered learning.

Using the AR approach, this study examined the impact of multiple active learning strategies as teaching interventions in the preclinical restorative dentistry course for third-year dental students. As shown in the previous literature, traditional learning methods, while effective, do not sufficiently support the students' learning. Furthermore, one teaching strategy alone does not provide adequate support for diverse student learning. 25 Hence, this study aspires to contribute to the limited extant literature on the implementation of mixed teaching methods to cover any gaps and weaknesses between the concept development of a particular practical procedure, its evaluation, and its practical implementation along with the evaluation from the peers.

By implementing various engagement and participation activities, we aim to improve the student learning experience and promote student-centered learning in dental education.

## Materials and Methods

### Study Design and Ethics

**Fig. 1:**
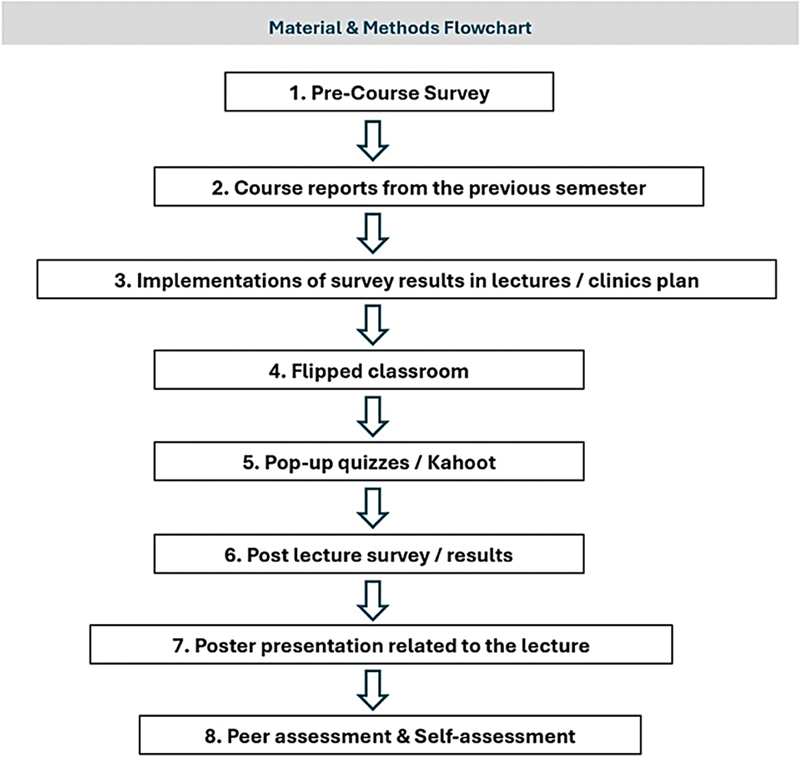
The flowchart shows the whole plan, starting from collecting the information to planning accordingly, to achieving the desired results.

This study employed an action-research methodology following the “Observation–Planning–Action–Reflection” (OPAR) cycle 26 over 10 weeks at the College of Dentistry, Imam Abdulrahman Bin Faisal University. Based on the study's main aim, the research design was chosen to rigorously examine the impact of multiple active learning strategies as teaching interventions in the course and their outcome based on peer/faculty assessment. The Institutional Review Board approved the study protocol (IRB-2023–02–216), and informed consent was obtained from all participants. The layout plan for the research is described in Fig. 1 .

### Study Population

The study included 58 third-year dental students (30 females, 28 males) enrolled in the Clinical Restorative Dentistry course, which focuses on the diagnosis, treatment planning, and execution of restorative procedures. While this study is based on a convenience sample of enrolled students, the sample size provides sufficient data for educational AR, as shown in similar studies. 27 28

### Research Implementation

The OPAR cycle consisted of four phases:

- Observation: review of previous course reports from the Vice Deanship of Academic Affairs.
- Planning: development of teaching strategy based on course objectives and pre- course survey results.
- Action: implementation of active learning interventions.
- Reflection: evaluation through surveys and assessments.
  The intervention included three components:
  - Flipped Classroom: pre-distributed materials and 15-minute mini-lectures with discussions.
  - Interactive Assessment: pop-up quizzes using Kahoot during lectures.
  - Poster Presentations: group presentations on composite cavity preparation techniques, evaluated by faculty using standardized rubrics.

### Data Collection and Assessment

Data were collected through two different aspects:

- Aspects that are the same for all study participants:
  - Poster presentation evaluations by three precalibrated faculty members using a standardized rubric ( Table 1 ).
  - Learning needs were identified through analysis of post-course feedback collected from the previous academic year's cohort.
  - Peer and self-assessment using the same standardized rubric.
- Aspects that were voluntary:
  - Post-lecture survey (16 items) evaluating teaching intervention ( Table 2 ).
  - Post-poster presentation survey (10 items) ( Table 3 ).

**Table 1:** The grading rubrics used to evaluate poster presentations

Areas of evaluation	0	1	2	3	4
Title	No title on project	Partial/incomplete project	The title is present but capitalized and spelled incorrectly	Titles are correctly written in neat and attractive letters	The title is correct, neat creative, and colorful
Knowledge	No knowledge of the poster	1 Knowledge is written, or knowledge is written incorrectly	2–3 Knowledge is written or/and partially correct	4 Knowledge is written and correct	5 Knowledge is written and correct
Objectives	No, or only 1 Objective is defined	1–2 Objectives are defined, or objectives wrongly defined	2–3 Objectives are defined, or objectives are wrongly defined	3–4 Objectives are defined, or objectives wrongly defined	All the objectives are defined, or objectives wrongly defined
Illustrations	No illustrations on the poster	1 Illustration related to the event	2 Illustrations related to the topic are present	3 Neat, clear illustrations related to the topic	4 Neat, clear, creative illustrations related to the topic
Appearance	The poster lacks neatness and is poorly organized	The poster lacks neatness and is mostly disorganized	The poster is somewhat organized and neat	The poster is organized and is neat and clear	The poster is very organized with neat, clear, easy-to-read lettering

**Table 2:** Showing post-lecture survey results for student participants

Questions asked for post-lecture survey	Mean ± SD
The attitude of the instructor was positive and friendly.	4.3 ± 0.8 a
Procedural value was clearly explained from the patient's perspective.	4.2 ± 0.8 a
The lecture was very interactive and involved everyone.	4.2 ± 0.8 a
Had a good grasp over the subject.	4.3 ± 0.7 a
The style of presentation and delivery of knowledge was excellent.	4.3 ± 0.7 a
Responsive to participants' questions.	4.3 ± 0.7 a
Maintained direction and control of session.	4.2 ± 0.7 a
The pace of the presentation was good.	4.1 ± 0.8 a
Had a good grasp over the subject.	4.2 ± 0.7 a
Discuss the information presented related to patients.	4.3 ± 0.6 a
The lecture enhanced my knowledge.	4.2 ± 0.7 a
Learning objectives were clearly met.	4.2 ± 0.8 a
Sessions progressed in a logical order.	4.1 ± 0.8 a
The contents were relevant, recent, and comprehensive.	4.2 ± 0.8 a
It was an excellent opportunity to learn new concepts.	4.3 ± 0.8 a
The information presented was relevant to the topic.	4.5 ± 0.6 a

Abbreviation: SD, standard deviation.

a Observed values of post-lecture survey questions are presented as mean and standard deviation.

**Table 3:** Showing post-poster survey results for student participants

Questions asked for post-poster survey	Mean ± SD
The topic was relevant to our need for knowledge and practice.	4.5 ± 0.6 a
The activity met my expectations.	4.1 ± 0.9 a
The activity was well organized.	3.7 ± 1.1
The experience was new and productive.	4.3 ± 0.8 a
The activity has a great impact on clinical practice and treatment planning.	3.9 ± 1.0
The objective of this activity was clear.	4.0 ± 1.1 a
This activity has enhanced my knowledge.	4.2 ± 0.9 a
Do you think other subjects/courses to have this type of activity?	3.7 ± 1.2
The interaction with the evaluators was fruitful and subjective.	4.0 ± 0.9 a
The experience of peer assessment with other colleagues was productive.	4.1 ± 0.9 a

a Observed values of post-poster survey questions are presented as mean and standard deviation.

Participation in the survey was voluntary through an electronic survey platform. Consent was obtained through link activation, where participants agreed to both study participation and research data use. The survey platform was configured to accept single responses per participant, ensuring data integrity. Participant confidentiality was maintained through anonymous data collection, and incomplete responses were excluded from statistical analysis. All surveys utilized a 5-point Likert scale (5 = strongly agree to 1 = strongly disagree). All survey instruments underwent a two-stage validation process. First, content validity was established through expert review by three dental education specialists at the College of Dentistry, who evaluated the questionnaire's content domains, clarity, and validity. Second, a pilot study was conducted with 20 students to assess readability, comprehension, and completion time. Pilot study participants were excluded from the final analysis to maintain data integrity.

Each poster presentation included a 10-minute presentation followed by a 5-minute Q&A session, with final grades calculated as the average of three precalibrated evaluators' scores based on a standardized rubric.

### Data Analysis

Along with observation and reflection throughout the process, as is customary in AR, quantitative analyses were performed on the post-lecture and post-poster survey data gathered for this study. These analyses entailed conducting a qualitative analysis of the data acquired through questionnaires, poster presentations, and observations made during and after the classroom study. The item responses on a 5-point numeric scale were presented as mean ± standard deviation by utilizing SPSS-27.0 (IBM product, Chicago, United States). These data were explored by using the Shapiro–Wilk test of normality, which revealed the data distribution to be normal. Cronbach's α was 0.91 overall for the surveys, showing high internal reliability. A one-sample *t* -test on post-test lecture survey and post-poster survey data was applied under the null hypothesis Ho: µ = 3.6, following 3.6 or above average response as high-quality performance. Comparative analysis of different assessment methods revealed interesting disparities between faculty and student evaluations, and peer assessment was performed by using an unpaired sample *t* -test. A *p* -value of less than or equal to 0.05 was considered a statistically significant result.

## Results

Using a four-phase AR approach, this research examined the impact of multiple active learning strategies as teaching interventions in the course. Data were collected from 58 third-year dental students through post-lecture and post-poster surveys, both utilizing a 5-point Likert scale (5 = strongly agree to 1 = strongly disagree). The post-lecture survey achieved a high response rate of 88% (51 participants), while the post-poster survey received a response rate of 66% (38 participants).

Analysis of the 16-item post-lecture survey demonstrated a high level of student satisfaction with the teaching intervention. The overall satisfaction rate for lecture organization and learning experience was 84% ( *M* = 4.2). Topic relevance emerged as the highest-rated aspect ( *M* = 4.5, 90%), while lecture pace and organization, though still favorable, received slightly lower ratings ( *M* = 4.1, 82%).

The 10-item post-poster survey revealed significant positive outcomes across multiple dimensions ( *p* < 0.05). Students expressed high satisfaction ( *M* = 4.1, 81%) with the poster presentation process and overall learning experience, particularly regarding relevance, expectations, productivity, objective achievement, knowledge enhancement, and peer assessment opportunities. The topic's relevance and practicality received the highest rating ( *M* = 4.5, 90%). However, some aspects showed nonsignificant results, including activity organization ( *M* = 3.7, 74%, *p* = 0.526), impact on clinical practice and treatment planning ( *p* = 0.072), and cross-course applicability ( *M* = 3.7, 74%, *p* = 0.671).

Comparative analysis of different assessment methods revealed interesting disparities between faculty and student evaluations, which was performed by using unpaired samples *t* -test. The faculty assessment panel awarded an average score of 3.5/5 for poster presentations, while the student self-assessment average was notably higher at 4.75/5 ( *p* < 0.001). Peer assessments showed slight gender-based variations, with male students awarding an average score of 4.5/5 and female students giving an average of 4.25/5 ( *p* = 0.886). These differences highlight varying perspectives on evaluation standards between faculty and students, as well as among students themselves.

## Discussion

Students typically valued the efficacy of inquiry-based instruction or instructional scaffolding that facilitates self-exploration, structured course materials that offer self-learning resources, experiential activities that enhance motivation, and collaborative learning opportunities that promote formative self-assessment. Active learning in mixed-methods teaching is effective when the instructor creates a motivating and engaging environment that encourages guided learning and self-exploration.

Implementing mixed teaching and learning methods in dental education comprises multiple domains and presents various challenges. 29 30 Our findings demonstrate the effectiveness of integrating active learning strategies through a flipped classroom model, continuous assessment, and poster presentations. Comparable outcomes were reported by Sivarajan et al, 31 32 who reported that the flipped classroom method and the formative assessment effectively transfer skills to the students quicker than traditional teaching methods.

The lack of a control group in this investigation was necessary. The performance of prior cohorts was used as a benchmark for comparison to guarantee a thorough assessment of the results. To create baseline performance indicators, data from prior years' students—including course reports from the previous 3 years and a brief questionnaire—were examined. This method made it possible to compare the present cohort with past data, which shed light on how well the course requirements were created.

The results showed that the flipped classroom approach significantly enhanced student engagement and interaction, consistent with previous research. 33 34 While most students responded positively to this teaching method, some expressed concerns about the nontraditional learning format, 35 and it also showed the synchronization with the previous studies, 36 37 where students familiar with conventional lectures may first oppose the flipped classroom model due to the transfer of learning responsibility to themselves, since they believe the workload would be excessively demanding.

These findings also establish the importance of careful implementation and student support while having some active teaching–learning strategy to be applied. Higher order thinking fostered through group discussions is key to the success of the flipped classroom approach. This method contrasts with traditional lecture-based learning, which primarily involves lower order thinking. 38 Our research similarly observed increased engagement, enhanced knowledge levels, and improved critical thinking skills by introducing flipped classroom strategies.

In the current study, gamification through Kahoot proved effective in maintaining student engagement and facilitating immediate feedback, supporting findings from previous studies. 39 40 Our post-lecture survey's high satisfaction rates (84%) suggest that interactive learning methods successfully transformed traditional lectures into engaging learning experiences.

By fostering effective involvement and helping students build self-information under the guidance of their teachers, creating posters during an activity and showing them to classmates can be said to support meaningful learning. This outcome is in line with the findings of studies conducted by Berry and Nyman, 41 Mulnix and Penhale, 42 Eker, 43 and Brown and Burroughs. 44

Through instruction centered on poster-based activities, the students gain a thorough understanding of a subject. Furthermore, compared with the other approaches, people are more successful and efficient in their endeavors when they are actively involved. 42 Incorporating game-based learning has been reported to generate excitement among students and teachers. This approach extends beyond the learning domain to include co-curricular and extracurricular activities. 39 The poster presentation component yielded particularly interesting results. Students demonstrated increased confidence and engagement, with significantly less anxiety compared with traditional presentations. 45 The dual requirement of presentation and Q&A sessions fostered deeper understanding and peer learning opportunities.

It has been demonstrated that expert evaluation is most beneficial when a student is evaluated by multiple instructors, each of whom is searching for a distinct component of the abilities being detailed. 46 47 The findings revealed a disparity between faculty members' perceptions of the quality and content of educational performance and those of the students. However, the disparity between faculty assessment scores (3.5/5) and student self-assessment scores (4.75/5) suggests a need for better alignment of evaluation criteria and expectations. Faculty members and students hold divergent views regarding the characteristics an exemplary faculty member should exhibit. The faculty and students also had differing opinions of the behaviors that faculty should exhibit. 48

Prichard and Ferreira 45 found that poster presentations offered significant advantages over traditional in-class presentations. Students delivering poster presentations showed lower anxiety levels and greater vocal participation compared with those giving traditional presentations. While poster presentations required students to manage both timed delivery and extensive Q&A sessions, this format appeared to foster better engagement and confidence. Our findings aligned with these observations, as students demonstrated strong preparation, organization, and confidence during their poster presentations. The combination of self-directed learning and immediate feedback through this format effectively supported students' professional development and clinical application skills.

The implementation of anonymous surveys at multiple stages provided valuable insights into student perceptions and learning experiences. 49 While most aspects received positive feedback, areas such as activity organization and clinical practice integration require further refinement.

The current research was supported by Hand and Rowe, emphasized on professional development and growth in the process shifted teachers' mindset from a “performance-based” approach to a “developmental approach,” and it was reaffirmed that the purpose of collecting student feedback was to support their continued improvement as teachers. 50 The feedback made it possible for teachers to participate freely in the process of getting student input and, using the data acquired, better address the needs of their students. 51 With appropriate professional development, student feedback can positively influence teaching practices. Moreover, participating in this activity can augment teachers' reflective abilities, provide insights into the unique needs of their pupils, and facilitate a discourse on teaching and learning within the classroom. 51

Several limitations should be considered when interpreting this study's findings. The study was conducted at a single institution with a relatively small sample size ( *n* = 58), with a lack of a control group for directly comparing learning outcomes between traditional and active learning methods, which may limit generalizability. The response rate for the post-poster survey (66%) was lower than the post-lecture survey (88%), potentially affecting the comprehensiveness of the feedback. Additionally, the study's 10 weeks may not have been sufficient to fully assess the long-term impact of these teaching interventions on clinical performance.

Thus, we recommend future studies that incorporate multiple institutions to increase sample size and diversity. Longitudinal studies would be valuable to evaluate the sustained impact of active learning strategies on clinical performance. Furthermore, the integration of clinical applications should be strengthened through case-based scenarios and direct patient care experiences. The implementation of a structured feedback system throughout the semester could help address organizational challenges identified in the poster presentations. Active learning, curricular integration, early clinical exposure, evidence-based teaching and evaluation, flipped classrooms, clinical-based learning, computer-assisted learning, and group discussions are encouraged across all courses in the dental curriculum.

## Conclusion

This study demonstrates that integrating active learning strategies through AR methodology can significantly enhance dental students' learning experience and achievement. The multi-strategy approach, in this study a combination of flipped classroom techniques, gamification, and poster presentations, effectively improved student engagement and practical application skills. However, the variation between faculty and student assessments highlights the need for the calibration of assessors using more standardized evaluation criteria.

Dental education ought to transition toward an integration type of teaching where they can learn theoretical knowledge practically along with their real-time assessments.
